# Developmental progression of lymphatic valve morphology and function

**DOI:** 10.3389/fcell.2024.1331291

**Published:** 2024-02-21

**Authors:** Michael J. Davis, Scott D. Zawieja, Ying Yang

**Affiliations:** ^1^ Department of Medical Pharmacology and Physiology, University of Missouri School of Medicine, Columbia, MO, United States; ^2^ Department of Molecular Pharmacology and Physiology, USF Health Morsani College of Medicine, University of South Florida, Tampa, FL, United States

**Keywords:** valve function, back leak, annulus, *Prox1*, 3D image, commissure, leaflet, confocal reconstruction

## Abstract

**Introduction:** The bileaflet valves found in collecting lymphatic vessels and some veins are essential for maintaining a unidirectional flow, which is important for lymphatic and venous function. Under an adverse pressure gradient, the two leaflets tightly overlap to prevent backflow. Valves are proposed to share four main stages of development, based on images obtained from randomly oriented valves in fixed mouse embryos, with the best structural views obtained from larger venous valves. It is not known at what stage lymphatic valves (LVs) become functional (e.g., able to oppose backflow), although a requirement for stage 4 is presumed.

**Methods:** To gain an insight into this sequence of events for LVs, we used *Prox1CreER* ^*T2*^ *:Foxo1* ^*fl/fl*^ mice and *Foxc2CreER* ^*T2*^ *:Foxo1* ^*fl/fl*^ mouse models, in which deletion of the valve repressor factor *Foxo1* promotes the development of new LVs in adult lymphatic vessels. Both strains also contained a *Prox1eGFP* reporter to image the lymphatic endothelium. Mesenteric collecting lymphatic vessels were dissected, cannulated, and pressurized for *ex vivo* tests of valve function. LVs at various stages (1–4 and intermediate) were identified in multi-valve segments, which were subsequently shortened to perform the backleak test on single valves. The GFP signal was then imaged at high magnification using a confocal microscope. Z-stack reconstructions enabled 1:1 comparisons of LV morphology with a quantitative measurement of back leak.

**Results:** As expected, LVs of stages 1–3 were completely leaky in response to outflow pressure elevation. Stage 4 valves were generally not leaky, but valve integrity depended on the Cre line used to induce new valve formation. A high percentage of valves at leaflet an intermediate stage (3.5), in which there was an insertion of a second commissure, but without proper luminal alignment, effectively resisted back leak when the outflow pressure was increased.

**Discussion:** Our findings represent the first 3D images of developing lymphatic valves and indicate that valves become competent between stages 3 and 4 of development.

## Introduction

A major function of the lymphatic system is to reabsorb excess interstitial fluid and protein and return them to the central veins. In humans, the daily transport of lymph can be as high as 12 L of fluid per day (Schmid-Schönbein, 1990; Wiig and Swartz, 2012). Because the Starling forces across the blood capillary wall favor net fluid filtration out of the vessels (Levick and Michel, 2010), the fluid not returned to the venous system accumulates in the interstitium (Chen et al., 1976; Aukland and Reed, 1993). Lymphatic capillary walls are composed of overlapping lymphatic endothelial cell (LEC) junctions, with high permeability, which allow reabsorption of interstitial fluid and protein (Baluk et al., 2007). Capillaries coalesce into precollecting and collecting lymphatic vessels, both of which contain bileaflet intraluminal valves, spaced every millimeter or so, to prevent backflow and ensure the movement of lymph centrally.

Lymphatic valve (LV) development occurs shortly after the formation of the lymphatic vasculature. Much of our knowledge of the LV developmental program comes from elegant studies using mouse models (Bazigou et al., 2009; Sabine et al., 2015), in which LVs begin to develop at E16.5 and mature around or shortly after birth. The amenability of the mouse model to genetic manipulation has been paramount to progress made in understanding of lymphatic valve development through the generation and use of fluorescent reporters, gene deletion, and fate mapping studies. The discovery of *Prox1* as a lymphatic endothelial specification factor and marker (Wigle and Oliver, 1999) and the development of a transgenic, tamoxifen-inducible *Prox1CreER* ^*T2*^ mouse model (Bazigou et al., 2011) to selectively excise engineered floxed alleles in LECs have led to identification of multiple genes critical for LV development, including *Foxc2*, *Gata2*, *Itga9*, *and Vegfr3* (Bazigou et al., 2009; Bazigou et al., 2011; Sabine et al., 2012; Liu et al., 2014; Kazenwadel et al., 2015). Many of the same genes are also critical for venous valve development (Bazigou et al., 2011). Lymphatic valve defects have been implicated as the underlying causes of many primary lymphedemas [see Table 1 in (Gonzalez-Loyola and Petrova, 2021; Iyer et al., 2020)] and understanding the stages of LV development could aid in further identification of the multiple factors critical for normal LV development.

Four stages of valvulogenesis have been proposed as follows (Bazigou et al., 2009; Bazigou et al., 2011; Sabine et al., 2012; Bazigou and Makinen, 2013; Bazigou et al., 2014): 1) emergence of a valve-forming cluster of endothelial cells (ECs), enriched in *Prox1*, *Gata2*, and *Foxc2*, which forms a ring-shaped valve territory around the vessel wall; 2) migration of those ECs into a circular shelf or annulus that protrudes into the vessel lumen; 3) elongation and downstream extension of ECs along one edge of the annulus to form a commissure in one wall of the vessel; and 4) downstream extension of ECs on the opposite side of the wall to form a second commissure. Diagrams presented in Sabine et al. (2012), Sabine et al. (2015) and Bazigou & Makinen (2013) elegantly depict various stages, although the transition from stages 3 to 4 is often abrupt. Due to the extremely small size of lymphatic vessels and LVs in developing mouse embryos, the best scanning electron microscopy images and 3D representations of valve structure are those presented in a study of larger, mouse venous valves at similar developmental stages (Bazigou et al., 2011). However, the stage at which LVs become fully functional remains unknown. Presumably, only fully mature (stage 4) LVs would be functional, but a recent study in which new LVs were induced to develop in the lymphatic networks of adult mice by deletion of the valve-repressing gene *Foxo1* (Scallan et al., 2021) suggests that LVs may become functional at an earlier stage. Scallan et al. (2021) found that new valves that were formed in the mesenteric lymphatics of adult *Foxc2CreER* ^*T2*^ *:Foxo1* ^*fl/fl*^ mice had no detectable levels of back leak even when tested under supraphysiological pressures, i.e., they were even more resistant to back leak than control valves in *WT* and *Foxo1* ^*fl/fl*^ mice. The valves selected for testing in that study were “mature,” i.e., having two functional leaflets, but their detailed anatomy was not characterized.

The goals of the present study were 1) to image the developmental stages of new LVs induced after *Foxo1* deletion in adult mice and 2) to correlate the structure of the valves at each stage with functional measurements of valve back leak performed on cannulated, *ex vivo* lymphatic segments containing single valves. Either *Prox1CreER* ^*T2*^ or *Foxc2CreER* ^*T2*^ was used to induce deletion of one or two alleles of the floxed *Foxo1* gene. A *Prox1eGFP* reporter was simultaneously expressed in the lymphatic endothelium so that fluorescence in the LEC layer could be imaged live and/or after fixation and immunostaining. The confocal image stacks of pressurized vessels with valves were reconstructed to generate the 3D images of the LVs at all stages and under favorable or adverse pressure gradients with open or closed leaflets. We hypothesized that LVs would become functional at a time intermediate between stage 3, when the first valve leaflet commissure forms, and stage 4, when the second commissure is fully formed. Our results show that 100% of LVs at stage 3.5 are indeed fully functional if induced by *Foxc2CreER* ^*T2*^, but only half of stage 3.5 valves induced by *Prox1CreER* ^*T2*^ are without back leak, with the rest leaking to varying degrees. This finding suggests that localization and/or titration of *Foxo1* levels may be critical for development of competent valves. While many stage 3.5 LVs are capable of preventing back leak, their off-axis orientation may alter the normal flow pattern of lymph and/or present an elevated resistance to flow in the open state, at least until stage 4 is reached.

## Methods

### Animal procedures

All procedures were approved by the Institutional Review Boards at the University of Missouri (#9797) and University of South Florida Morsani College of Medicine (#10146) and complied with the standards stated in the “Guide for the Care and Use of Laboratory Animals” (National Institutes of Health, revised 2011).

### Mice

*Prox1eGFP* mice were a gift from Young-Kwon Hong, University of Southern California. *Prox1CreER* ^*T2*^ mice were a gift from Taija Mäkinen, Uppsala University. *Foxc2CreER* ^*T2*^ mice, retaining one functional *Foxc2* allele, were a gift from Sathish Srinivasan, Oklahoma Medical Research Foundation. *Foxo1* ^*fl/fl*^ mice (Scallan et al., 2021) were crossed with *Prox1CreER* ^*T2*^ to generate *Prox1CreER* ^*T2*^ *:Foxo1* ^*fl/fl*^ and *Prox1CreER* ^*T2*^ *:Foxo1* ^*+/fl*^ mice or with *Foxc2CreER* ^*T2*^ mice to generate *Foxc2CreER* ^*T2*^ *:Foxo1* ^*fl/fl*^ mice. *Prox1eGFP* (Choi et al., 2011) was bred into both strains. For genotyping, genomic DNA was extracted from tail clips using the HotSHOT method. Genotypes were determined by PCR with 2x M-PCR OPTI Mix (Catalog # B45012, Bimake, Houston, TX) according to the provider’s instructions. Both male and female mice were used for all protocols. For postnatal deletion, mice were induced with two subcutaneous injections of 100 mg tamoxifen (20 mg/mL; safflower oil) for *Prox1CreER* ^*T2*^ on P (postnatal) 1 and P3 and three subcutaneous injections of 100 mg tamoxifen for *Foxc2CreER* ^*T2*^ on P1, P3, and P5. *Prox1CreER* ^*T2*^ *:Foxo1* ^*fl/fl*^, *Prox1CreER* ^*T2*^ *:Foxo1* ^*+/fl*^, and *Foxc2CreER* ^*T2*^ *:Foxo1* ^*fl/fl*^ mice were bred and induced at the University of South Florida and then shipped to the University of Missouri for functional tests and image analysis.

### Vessel isolation, cannulation, and pressure control

Mice were anesthetized with ketamine/xylazine (100/10 mg/kg, i.p.) and placed face up on a heated tissue dissection pad. The abdomen was opened with an incision, and the entire small intestine was removed, rinsed, and pinned to a Sylgard-coated 60-mm tissue culture dish filled with Krebs–albumin solution. Individual mesenteric collectors (usually from the duodenum or jejunum) were excised with minimal attached fat and pinned to a dissection chamber using a 40-μm wire. After removing more fat and connective tissue, vessels containing 3–4 valves at various stages of development were then transferred to a 3-mL myograph chamber containing Krebs–albumin solution. Each vessel was cannulated at each end with a glass micropipette (40–50 μm OD tip), pressurized, and further cleaned. The chamber assembly, attached micropipettes, pipette holders, and micromanipulators were transferred to the stage of an inverted microscope. Polyethylene tubing connected the back of each micropipette to a low-pressure transducer and a computerized pressure controller (Microcirculation Research Institute, Texas A&M University), allowing independent control of inflow (Pin) and outflow (Pout) pressures, as described previously (Davis et al., 2011; Davis et al., 2023). Valve test protocols were performed at 37°C in Ca^2+^-free Krebs buffer to prevent pressure fluctuations that otherwise could have interfered with valve function tests. A constant exchange of buffer at a rate of 0.5 mL/min was maintained by using a peristaltic pump (MINIPULS 3, Gilson). Custom LabVIEW programs [(Davis, 2023b); National Instruments; Austin, TX] acquired analog data from the pressure transducer amplifiers (CyQ Instr., Nicholsville, KY) at 30 fps simultaneously with vessel inner diameter, which was detected from video images (Davis, 2005) acquired from a Basler FireWire camera (scA640-70 fm). Digital videos of the valve function protocols, with embedded pressure data, were recorded for additional off-line analyses using a custom LabVIEW program (Davis, 2023a).

### Valve function tests

After a multi-valve segment was mounted on the microscope, brightfield images were collected, and estimates of the various valve stages were made. The segment was then shortened to a single valve for functional tests, and the remainder of the segment was stored at room temperature for later re-cannulation and study that same day. In the one-valve segment, an ∼10-μm initial hole was made with a pilot micropipette, which was then withdrawn and replaced with a servo-null micropipette (Instrumentation for Physiology and Medicine, San Diego, CA) to measure the luminal pressure (Psn) on the inflow side of the valve. After insertion, the servo-null micropipette (tip diameter = 3–5 μm) was advanced so that the tapered shank sealed the hole. The Psn measurement was then calibrated by adjusting the gain and offset of the amplifier/computer while increasing Pin and Pout simultaneously between 0.5 and 10 cmH_2_O, respectively (Davis et al., 2023). Not all valves could be studied: some were spaced too close together to allow cannulation and subsequent insertion of a servo-null pipette.

To measure back leak through a closed valve, Pin and Pout were set to 0.5 cmH_2_O with the valve open, and Pout was increased to 10 cmH_2_O, ramp-wise, over a 35-s period, while Pin was held at 0.5 cmH_2_O. An outflow pressure ramp to 10 cmH_2_O is the standard protocol that we have used in previous studies on valve function (Sabine et al., 2015; Lapinski et al., 2017; Munger et al., 2017; Davis et al., 2022) and that the pressure level is needed to detect subtle-to-intermediate valve leaks that are often observed both in *WT* and knock-out mouse models. Normal valves closed as Pout exceeded ∼1 cmH_2_O and remained closed for the duration of the Pout ramp. Pressure back leak through the closed valve was measured with the Psn micropipette, which could resolve changes as small as ∼0.05 cmH_2_O on the inflow side of the valve. This test was repeated three times, and the values of back leak (Psn–Pin) were averaged to obtain a single representative point for each valve. Values of back leak were not corrected for pressure drops across the cannulating pipettes, but the same set of Pin and Pout pipettes were used throughout this study to ensure consistency in the back leak measurements between vessels.

### Classification of valve developmental stages

Each valve was tentatively classified as stages 1, 2, 3, or 4 under brightfield illumination before performing the functional tests of back leak. In some cases, the stage was subsequently confirmed or reclassified after confocal imaging.

### Confocal imaging and immunostaining

Previously, we found that a higher adverse pressure gradient was required to close a given valve after fixation, such that the leaflets sometimes no longer overlapped properly, suggesting that fixation altered the normal leaflet properties (Davis et al., 2023). For this reason, some vessels were imaged after valve function tests but before fixation so that views of the open and closed states could be obtained. In these cases, the segment was removed, transferred to a different chamber, recannulated in Ca^2+^-free Krebs buffer, and imaged on the confocal microscope at room temperature. In other cases, the vessel was fixed immediately while cannulated and pressurized to maintain an open lumen and stored in PBS at 4°C for later immunostaining and imaging. Shortening vessels to test individual valves often required discarding some valves that were too closely spaced to study. In general, only every other valve could be subjected to functional tests. To gain a perspective on the number of valves occurring at different stages in a particular genotype (*Foxo1* ^*fl/fl*^, *Prox1CreER* ^*T2*^ *:Foxo1* ^*fl/f*^ *, Prox1CreER* ^*T2*^ *:Foxo1* ^*+/fl*^, and *Foxc2CreER* ^*T2*^ *:Foxo1* ^*fl/fl*^ mice), a few long segments from each genotype (mostly unbranched or with sealed branches) were dissected, cleaned, and cannulated and either imaged immediately on the confocal microscope or fixed while pressurized and then stored in PBS at 4°C for later immunostaining and imaging.

A Leica DMi8 inverted fluorescence microscope with an Andor Dragonfly 200 spinning disc confocal imaging platform was used to image GFP fluorescence in the LEC layer. Confocal stacks were collected with a 25x or 40x water objective and then subjected to deconvolution and reconstructed into a 3D image. To visualize the open valve, Pin was set higher than Pout after which the pressures were reversed to image the closed valve (if it closed). After live imaging, the vessel was then fixed in 1% PFA for 20 min while pressurized and stored in 1% PFA overnight before rinsing five times in PBS and stored in PBS for later immunostaining and re-imaging.

For imaging of GFP and other LEC markers in fixed vessels, the vessels were washed three times in PBS, permeabilized with a 0.1% Triton X-100 solution in PBS for 1 h, blocked for non-specific binding using BlockAid solution (Thermo Fisher^®^ B10710) for 2 h at 4°C, and then incubated at 4°C overnight with primary antibodies. The antibodies were anti-GFP (Invitrogen #A-11122; 1:200), anti-CD144 (VE-cadherin, BD PharMingen #550548; 1:100), and anti-smooth muscle α-actin (clone 1A4, SMA A2547), Sigma; 1:500) in BlockAid solution. Vessels were washed for 2–4 h in PBS at 4°C, replacing PBS three to four times over the washing period. Vessels were then incubated overnight with secondary antibodies (donkey anti-mouse, anti-rabbit, and donkey anti-rat at 1:200 dilution, Invitrogen #A32787, #A21206, and #A48272, respectively) at 4°C. The vessels were washed with PBS for 2 h on a rocker platform replacing the PBS solution every ∼30 min and then incubated in PBS with NucBlue™ Live ReadyProbes™ reagent (Hoechst 33342) (Invitrogen Cat. No. R37605) as per the manufacturer’s instructions. Finally, the vessels were transferred to a myograph chamber, where they were re-cannulated and pressurized in PBS in an observation chamber with a #1.5 coverslip bottom. Fluorescence image stacks were acquired on the confocal imaging platform using a 40-μm pinhole disk, with Borealis™ enhanced illumination. Whole-valve or half-valve images were acquired with a Zyla PLUS 4.2 Megapixel sCMOS camera and a Leica 25x water objective HC FLUOTAR L 25x/0.95 W VISIR or a HCX PL APO 40x/1.10 W CORR objective using excitation wavelengths from 405-nm, 488-nm, 561-nm, and 647-nm solid-state diode laser lines. *Z*-axis image-stacks were acquired in 0.5-µm intervals for the 25x objective and 0.24 µm intervals for the 40x objective and then processed and rendered using Imaris x64 9.7.2, as three-dimensional orthographic isometric projections.

### Solutions and chemicals

Krebs buffer contained 146.9 mM NaCl, 4.7 mM KCl, 2 mM CaCl_2_·2H_2_O, 1.2 mM MgSO_4_, 1.2 mM NaH_2_PO_4_·H_2_O, 3 mM NaHCO_3_, 1.5 mM Na-HEPES, and 5 mM D-glucose (pH = 7.4). A buffer of the same composition (“Krebs-albumin”) also contained 0.5% bovine serum albumin. The Krebs-albumin solution was present both luminally and abluminally during cannulation, and the abluminal solution was constantly exchanged with Ca^2+^-free Krebs solution during the experimental protocol. The Ca^2+^-free Krebs solution contained 3 mM EGTA instead of CaCl_2_·2H_2_O. Purified BSA was obtained from US Biochemicals (Cleveland, OH), MgSO_4_ and Na-HEPES were obtained from Thermo Fisher Scientific (Pittsburgh, PA), and all other chemicals were obtained from Sigma-Aldrich (St. Louis, MO), except as otherwise specified.

### Statistics

Microsoft Excel was used to compile the initial data and to calculate back leak (Pout–Psn) at Pout = 10 cmH_2_O. Igor (WaveMetrics, Lake Oswego, OR) was used for the display of representative traces. Prism v9 (GraphPad, San Diego, CA) was used for summary plots and statistics. Specific statistical tests are stated in the figure legends.

## Results

### 3D images of mature valves

**FIGURE 1:**
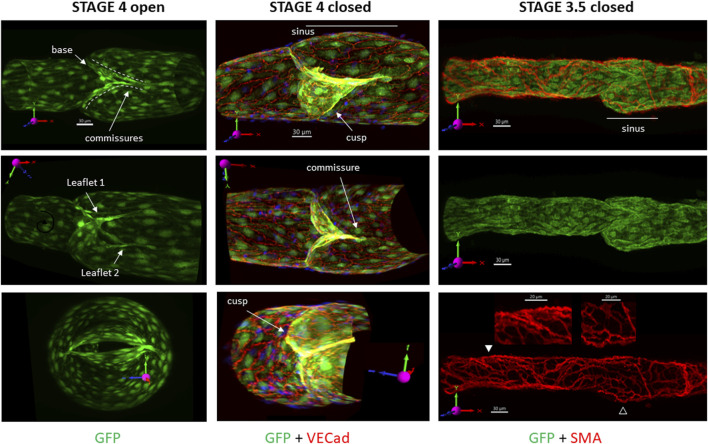
3D images of mature valves. The top images show the respective valves as viewed under the confocal microscope, without rotation. The first row of images provides the projection acquired from imaging (not rotated off the imaging axis) and its corresponding scale bar, which is omitted from the subsequent images when the vessel is rotated off the native axis. The vessels are oriented in such a way that the X-axis on the 3D compass in the image is pointing in the direction of flow and is maintained in all images to assist in visualization. The same convention applies to the top images in the other figures. Column 1: GFP signal only, showing three different positions of an open stage 4 valve (under a forward pressure gradient) from a *Foxo1* ^*fl/fl*^ mouse. Labels indicate the various terms used subsequently. Column 2: GFP labeling of LECs (green) with overlay of VE cadherin (red) of a stage 4 valve in a *Prox1CreER* ^*T2*^ *:Foxo1* ^*fl/fl*^ vessel held under an adverse pressure gradient. Only half of the confocal image stack is shown. Column 3: GFP labeling of LECs (green) and smooth muscle cells (red) in a not-quite mature valve from a *Foxo1* ^*fl/fl*^ mouse, showing a prominent sinus region on the lower side and reduced density of LMC coverage in that sinus (open arrowhead) compared to the tubular region (filled arrowhead). The middle image is a GFP signal only; the bottom image is an SMA signal only, with zoomed regions corresponding to the arrowheads. See Supplementary Movies S1–3 for rotatable 3D views of each of these valves.

Views of reconstructed and deconvolved confocal image stacks of GFP fluorescence in a stage 4 LV are shown in column 1 of Figure 1. The slightly off-axis view of the valve in the middle image clearly shows the shapes of the two leaflets, the base of the valve, and the commissures along which the leaflets are inserted into the wall. The open valve is shown end-on in the lower panel. Column 2 shows another stage 4 valve under an adverse pressure gradient sufficient to close the valve. In addition to the GFP signal in the LECs (green channel), VE cadherin labeling is shown in the red channel (yellow = overlap). A prominent sinus is visible (the viewing angle partially obscures the sinus on the other side). Column 3 shows a nearly mature valve with GFP fluorescence in the green channel and SMA staining of the LMCs in the red channel. Note the prominent valve sinus on the lower side (middle panel) and the lower density of LMCs in the sinus region (closed arrowheads, lower panel) compared to the tubular part of the vessel (open arrowheads). We refer to this valve as stage 3.5 for reasons discussed below. Supplementary Movies 1–3 provide rotatable 3D views of the three respective valves.

### Imaging LVs at various developmental stages

Initial imaging studies of developing LVs induced by *Foxo1* deletion revealed categories of LVs very similar to the four stages described for venous valve development by Bazigou *et al.* (2011). The ability to cannulate lymphatic collectors from adult mice, pressurize and rotate them as needed, and then collect the confocal z-stack images of the entire vessel allowed us to visualize the LVs in three dimensions at their various developmental stages.

**FIGURE 2:**
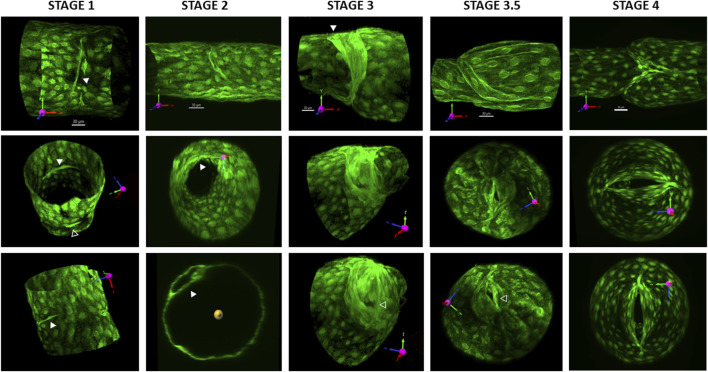
3D reconstructions of valves at various stages. All valves are from *Prox1CreER* ^*T2*^ *:Foxo1* ^*fl/fl*^ vessels and were imaged 2–4 weeks after tamoxifen induction. GFP was either imaged live with the vessel pressurized or after fixing the vessel and staining for GFP. The first row of images provides the projection acquired from imaging (without rotation) and its corresponding scale bar. See the explanation in Figure 1. Column 1 shows three different views of a stage 1 valve. In the top panel, the solid white arrowhead points to a band of condensed LECs. The middle and lower panels show two different views of the same bands after rotation to different angles. In the middle panel, the open white arrowhead points to a shorter band of condensed LECs on the opposite side of the vessel. Column 2 shows three different views of an early stage 2 annulus that projects into the lumen. The solid arrowheads in the middle and lower panels point to the LEC shelf. The lower panel is an orthogonal projection, showing the thickness of the shelf. Column 3 shows three different views of a stage 3 valve. The solid white arrowhead in the top panel points to the region of one wall where the annulus has attached and started to form a commissure. The open arrowhead in the lower panel indicates the part of the opening of the valve in an end-on view. The fourth column shows three different views of a stage 3.5 valve. Note how the valve opening is close to one wall of the vessel (open arrowhead in the bottom panel). The fifth panel shows three different views of a mature, stage 4 valve. The lower two panels show how the valve opening is oriented in the centerline of the vessel lumen. See Supplementary Movies S4–8 for rotatable 3D views of each of these five respective valves.

Stage 1 LVs consisted of a band of condensed LECs that partially or fully encircled the circumference of the lymphatic vessel. Because very few complete stage 1 rings remained after the time that elapsed between tamoxifen induction at USF and valve studies at MU, we found only a few complete rings and were unable to obtain their confocal image stacks. The LV in the first column of Figure 2 is a partial ring. The first column shows three different views of the stage 1 valve. In the top panel, the vessel is in the native image acquisition axis with the x-axis scaled and oriented in the normal direction of the flow. A few LECs have condensed into a band that encircles ∼ one-fourth of the vessel circumference (solid white arrowhead). The middle and lower panels show two different views of the same bands after rotation to different angles. A shorter band of condensed LECs is evident on the opposite side of the vessel (open white arrowhead in the middle panel). Supplementary Movie S4 provides a 3D rotation view of this stage 1 valve.

Images provided in the second column of Figure 2 show three different views of a stage 2 valve, which is beginning to form an annulus that projects into the lumen. LVs at stage 2 were consistent with previous descriptions of LECs that had migrated into the vessel lumen to form a circular shelf that eventually extends into an annulus, encircling and restricting the lumen. The width of the LEC shelf, which determined the size of the luminal opening, was variable. Many annuli were oriented perpendicular to the flow axis, but a few were slightly angled at the time of study, suggesting they were transitioning to stage 3. The top panel shows the annulus from the side, bending in the direction of an applied forward pressure gradient. The middle panel shows an end-on view, with the partial shelf protruding into the vessel lumen. The lower panel is an orthogonal projection showing the thickness of the forming shelf. Supplementary Movie S5 provides a 3D rotation view of this stage 2 valve.

Images in the third column of Figure 2 show three different views of a stage 3 valve. At stage 3, LECs on one side of the annulus have attached and migrated downstream and started to form a commissure in the wall (solid white arrowhead). The valve remained opened regardless of the direction of the applied pressure gradient. The open arrowhead in the lower panel points to the opening in an end-on view. Supplementary Movie S6 provides a 3D rotation view of this stage 3 valve.

**FIGURE 3:**
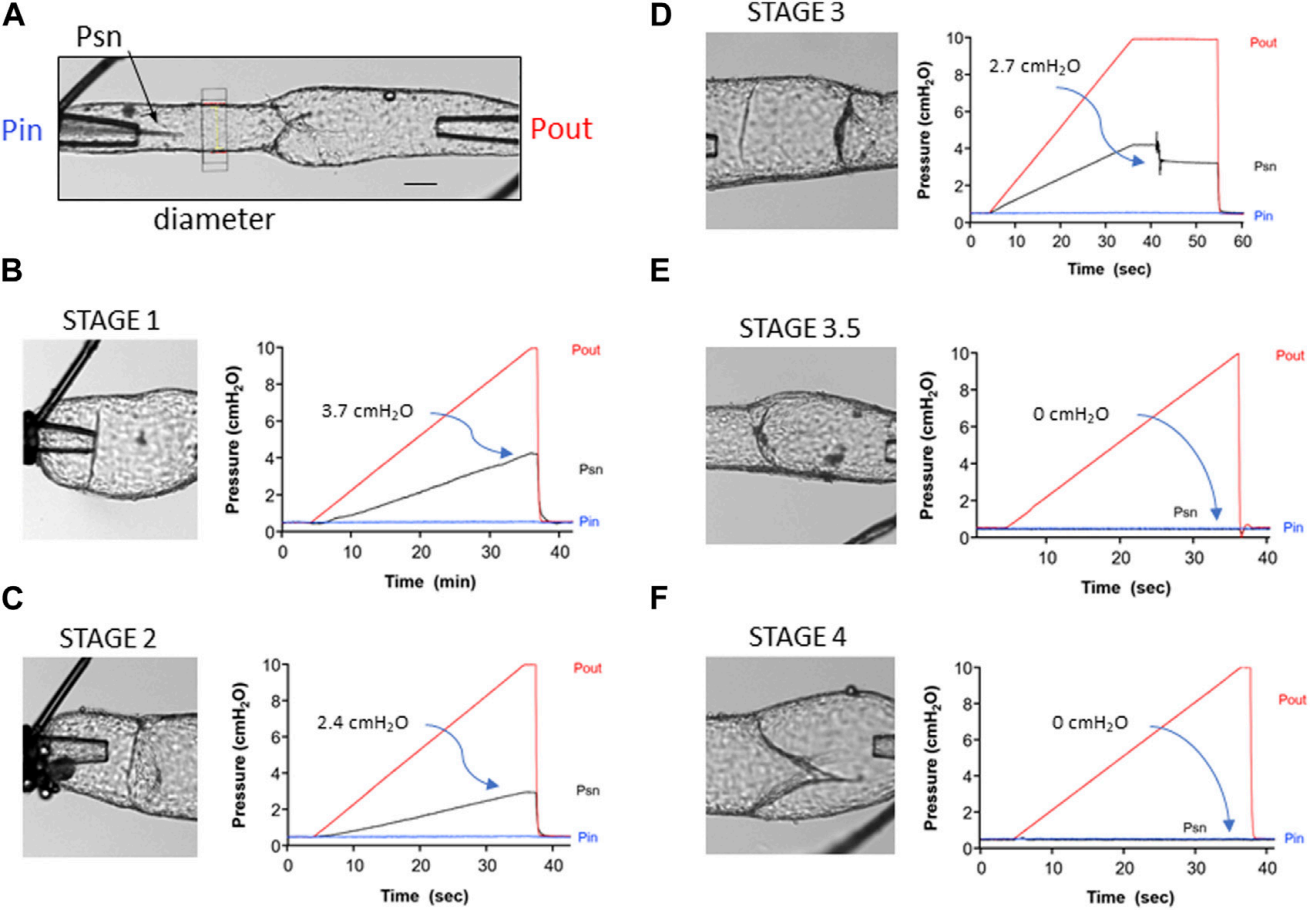
Representative traces showing the back leak test protocol for the different stages of developing valves. **(A)** Diagram of the test system showing the Pin and Pout cannulating pipettes, the position of the diameter tracking window and of the tip of the servo-null (Psn) pipette positioned upstream from the valve. Calibration bar = 50 μm. **(B)** Back leak test on a stage 1 valve. Pin was held at 0.5 cmH_2_O, while Pout was increased ramp-wise from 0.5 to 10 cmH_2_O. The “valve” offered no resistance to backflow, such that at the end of the ramp, Psn reached 4.2 cmH_2_O. Back leak (Psn–Pin) = 3.7 cmH_2_O. **(C)** Back leak test for a stage 2 valve (annulus). **(D)** Back leak test for a stage 3 valve that moved and partially closed after Pout had reached 10 cmH_2_O. **(E)** Back leak test for a stage 3.5 valve that was completely competent (back leak = 0 cmH_2_O). **(F)** Back leak test for a stage 4 valve that was normal (back leak = 0 cmH_2_O).

We observed what appeared to be a stage intermediate between stages 3 and 4, which we termed stage 3.5. Images in the fourth column of Figure 2 show three different views of a stage 3.5 valve. At this stage, the opposite side of the annulus had begun to migrate downstream to the extent that the “LEC shelves,” which would eventually form the leaflets, were sufficiently flexible at their tips that the structure could deform and seal if the appropriate adverse pressure gradient was applied, potentially preventing backflow. These stage 3.5 valves were not yet fully mature, i.e., with their leaflets oriented in the middle of the flow stream, but instead were oriented with the opening always canted toward one wall of the vessel. The openings were visible in confocal reconstructions but were usually difficult to visualize in brightfield illumination mode, making stages 3.5 and 4 often difficult to distinguish under brightfield illumination. Sinuses evident at stage 3.5 were limited to the side on which the commissure had been fully inserted (lower side of the top panel); Figures 3E, 5). Supplementary Movie S7 provides a 3D rotation view of this stage 3 valve.

In the fifth column of Figure 2, three different views of a stage 4 valve are shown. Stage 4 valves were defined as fully mature, bicuspid valves in which both leaflets were aligned with the flow stream in the center of the lumen. Most sinuses associated with stage 4 valves were symmetrical (top panel). The lower two panels show the valve opening oriented in the centerline of the vessel lumen. A movie showing 3D rotation of this structure is provided in Supplementary Movie S8.

### Tests of back leak at different developmental stages

Back leak tests were performed for LVs at various stages of development. Valves were tentatively classified as stages 1, 2, 3, or 4 prior to functional testing, based on their appearance under brightfield microscopy after cannulation and pressurization. Stage 1 valves were clear rings or partial rings. Stage 2 valves were identified after bending or turning the vessel on the pipettes to view, if possible, the valve from the luminal axis to confirm the presence of an annulus. Manipulation of the prevailing pressure gradient would cause the annulus of stage 2 valves to protrude in either direction. In stage 3 valves, one edge had migrated downstream from the other, giving the valve an angled appearance relative to the normal flow direction; the leaflet was not usually visible while changing the pressure gradient. Stage 4 valves were characterized by having two leaflets that would each flex as the pressure gradient was altered.

Figure 3A depicts the experimental configuration of the inflow, outflow, and servo-null micropipettes relative to the position of the valve prior to the start of a valve test. The servo-null pipette measured the pressure (Psn) between the valve and the inflow pipette and was a sensitive indicator of back leak, as documented in previous publications (Sabine et al., 2015; Lapinski et al., 2017; Scallan et al., 2021). Diameter measurement on the inflow side of the valve provided confirmation of back leak; however, diameter traces are not shown in order to simplify the figure. Panels B–F show the brightfield images of each valve (left side) and the various pressures (Pin, Pout, and Psn) as a function of time (right side) during the back leak test. An example of a back leak test for a stage 1 valve is shown in Figure 3B. The image shows that this structure is only a partial ring. As predicted, the “valve site” was completely leaky, such that Psn increased to a value of 4.2 cmH_2_O as Pout was increased ramp-wise from 0.5 to 10 cmH_2_O (the theoretical limit is ∼4.75 cmH_2_O, a value half-way between that of Pout and Pin at the end of the ramp, with some variation depending on the resistances of Pin and Pout pipettes and relative positions of the three pipettes with respect to the valve). The value of back leak (3.7 cmH_2_O) shown on the graph is the difference between Psn and Pin when the Pout reached 10 cmH_2_O. An example of a back leak test for a stage 2 valve is shown in Figure 3C. Back leak was somewhat lower than maximal (2.4 cmH_2_O) as the narrow annulus offered partial resistance to backflow when the Pout was increased. An example of a back leak test for a stage 3 valve is shown in Figure 3D. This valve was unusual, in that it was completely leaky until Pout reached 10 cmH_2_O, and then the leaflet suddenly moved backward, offering partial resistance to backflow; steady-state back leak was 2.7 cmH_2_O. A stage 1 valve is also present to the left of the valve being tested but did not interfere with the back leak test of the stage 3 valve. An example of a back leak test for a stage 3.5 valve is shown in Figure 3E. Although this valve did not look fully mature, the leaflets closed effectively when Pout was elevated and prevented back leak (0 cmH_2_O). Not all stage 3.5 valves behaved in this manner, as revealed by a plot of the individual back leak values for all stage 3.5 valves in the next figure. A partial sinus is also evident on the upper side of the vessel where the commissure has inserted. An example of a back leak test for a stage 4 valve is shown in Figure 3F. This was a typical, fully mature valve, with the commissures for both leaflets oriented along the midline of the vessel, and with a distinct, symmetrical sinus (the lower half is partially obscured in this angle of view). Back leak for this valve was 0 cmH_2_O at all levels of Pout.

### Correlation of function with the developmental stage

**FIGURE 4:**
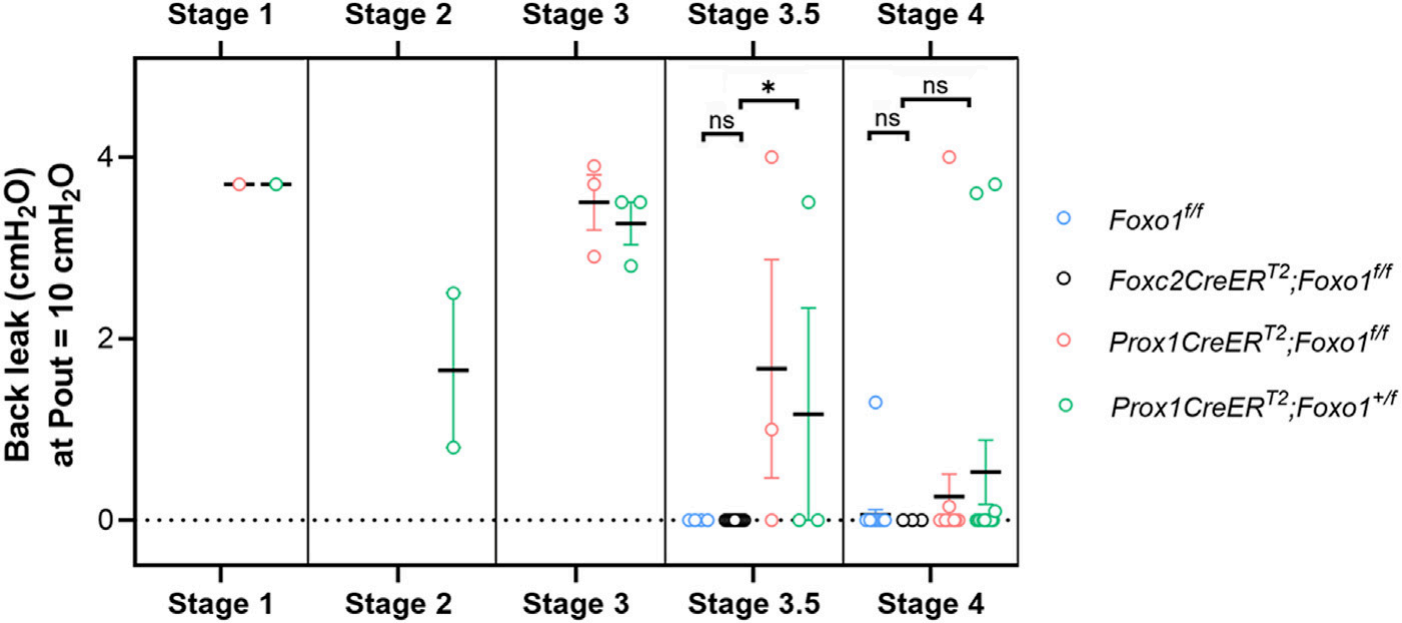
Summary data for back leak measured in valves at different stages. *Foxc2CreER* ^*T2*^ *:Foxo1* ^*fl/fl*^ data are reproduced from (Scallan et al., 2021), but split into stages 3.5 and 4. *Foxc2CreER* ^*T2*^ *:Foxo1* ^*fl/fl*^ valves were not studied at stages 1–3. n = 26 for *Foxo1* ^*fl/fl*^; n = 12 for *Foxc2CreER* ^*T2*^ *:Foxo1* ^*fl/f*l^; n = 23 for *Prox1CreER* ^*T2*^ *:Foxo1* ^*fl/fl*^; n = 23 for *Prox1CreER* ^*T2*^ *:Foxo1* ^*+/fl*^ *.* Differences between *Foxo1* ^*fl/fl*^, *Foxc2CreER* ^*T2*^ *:Foxo1* ^*fl/fl*^, and *Prox1CreER* ^*T2*^ (with *Foxo1* ^*fl/fl*^ and *Foxo1* ^*+/fl*^ combined due to low numbers in each group) for stage 3.5 and stage 4 valves were tested using Kruskal–Wallis non-parametric tests; * = *p* < 0.05, ns = not significant.

The values of back leak (measured at Pout = 10 cmH_2_O) are shown for 84 valves from four genotypes of mice and are grouped according to the developmental stage (Figure 4). For reasons previously stated, the limited resolution of structures under brightfield microscopy resulted in a few stage 3 and 3.5 valves being confused. In some of these cases, the classification of the valve stage was changed after observing the valve anatomy in more detail during confocal imaging. *Foxo1* ^*fl/fl*^ valves (blue circles) served as the control valves, and only stage 3.5–4 valves were observed in this genotype. All *Foxo1* ^*fl/fl*^ valves (*n* = 26), with one exception, had essentially no back leak. The data for *Foxc2CreER* ^*T2*^ *:Foxo1* ^*fl/fl*^ valves (black circles) were reproduced from a recent study (Scallan et al., 2021), except that for the present purposes, the valves were split into stage 3.5 (9 of 12) and stage 4 (3 of 12) valves according to brightfield images obtained at that time. No back leak was detected in any stage 3.5 or stage 4 *Foxc2CreER* ^*T2*^ *:Foxo1* ^*fl/fl*^ valves. Stage 1–3 valves were presumably present in some of the *Foxc2CreER* ^*T2*^ *:Foxo1* ^*fl/fl*^ vessels, but those were not imaged or studied. Back leak values for *Prox1CreER* ^*T2*^ *:Foxo1* ^*fl/fl*^ and *Prox1CreER* ^*T2*^ *:Foxo1* ^*+/fl*^ (haplodeficient in *Foxo1*) mice are shown in red and green circles, respectively. All stage 3 *Prox1CreER* ^*T2*^ valves (both *Prox1CreER* ^*T2*^ *:Foxo1* ^*fl/fl*^ and *Prox1CreER* ^*T2*^ *:Foxo1* ^*+/fl*^) were highly leaky. Half (3 of 6) of stage 3.5 *Prox1CreER* ^*T2*^ valves were tight, and the rest had varying degrees of leak. The majority of stage 4 *Prox1CreER* ^*T2*^ valves were tight, but 3 of 28 were highly leaky.

### Imaging open and closed valves at stages 3.5 and 4

**FIGURE 5:**
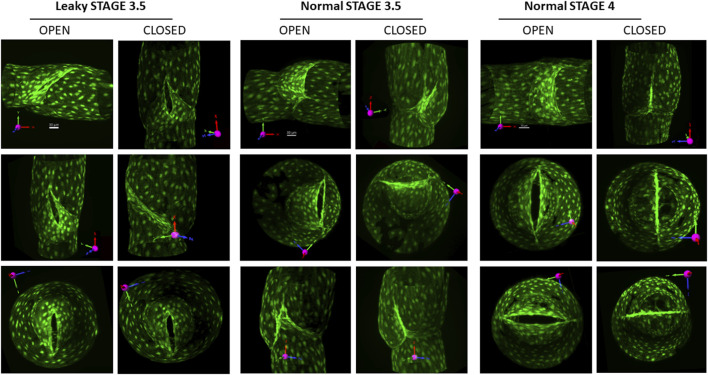
Views of stage 3.5 and 4 valves in open and closed positions. To open the valve, a forward pressure gradient ∼5 cmH_2_O was applied; to close the valve, the gradient was reversed. Columns 1–2: three views of an open and closed defective stage 3.5 valve (the opening narrowed slightly, but never closed under an adverse pressure gradient). Columns 3–4: three views of an open and closed stage 3.5 valve that had no back leak when closed. Columns 5–6: three views of a normal stage 4 valve with no back leak when closed. The first row of images provides the projection acquired from imaging (without rotation) and its corresponding scale bar. See the explanation in Figure 1.

Several valves at the more advanced stages were imaged without fixation, with favorable or adverse pressure gradients applied to view the valves in their open and closed states, respectively. Examples of three such valves are shown in Figure 5. The first two columns show a leaky stage 3.5 valve from a *Prox1CreER* ^*T2*^ *:Foxo1* ^*fl/fl*^ *mouse*, which never completely closed even in the face of an adverse pressure gradient of ∼5 cmH_2_O. The opening is evident in the lower panel of column 2. Columns 3–4 show a different stage 3.5 valve from a *Prox1CreER* ^*T2*^ *:Foxo1* ^*fl/fl*^ mouse, which closed when an adverse pressure gradient was applied (Column 4). The off-centerline orientation of the valve opening is clear in these images. Columns 5–6 show a stage 4 valve from a *Prox1CreER* ^*T2*^ *:Foxo1* ^*fl/fl*^ mouse that opened and closed normally with the application of a favorable (column 5) or adverse pressure gradient (column 6). Note that the opening of the stage 4 valve is aligned with the vessel centerline, and its opening nearly spans the width of the lumen, thus providing a larger surface area than that of the stage 3.5 valve.

### Leaky stage 4 valves

**FIGURE 6:**
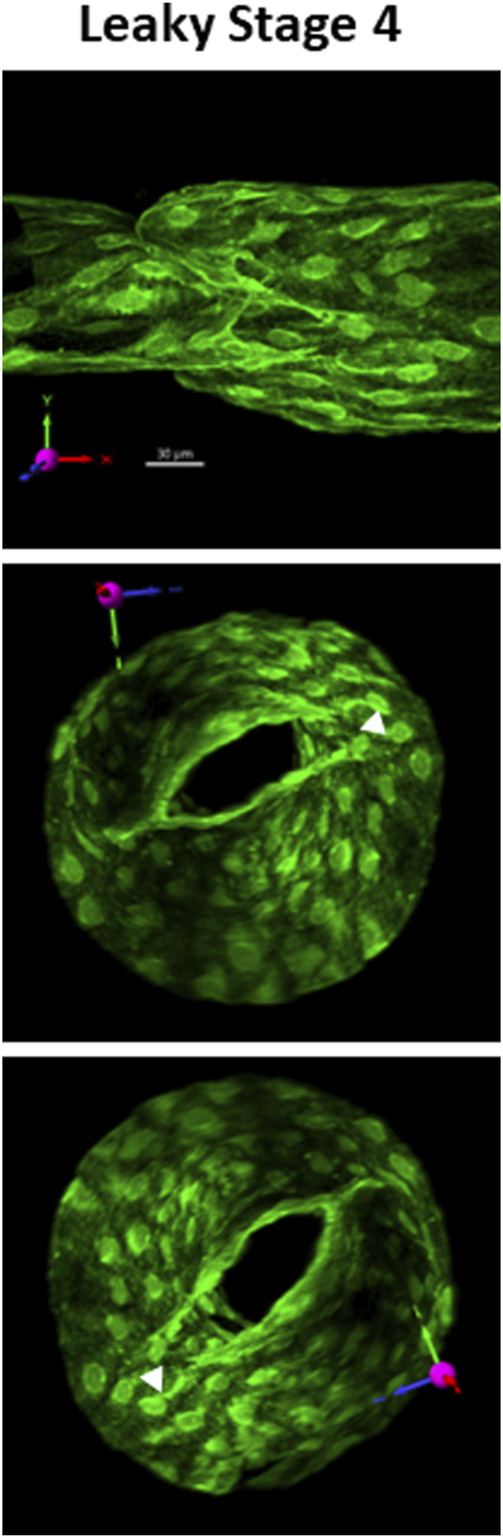
Example of a leaky stage 4 valve. Three views of a defective stage 4 valve from a *Prox1CreER* ^*T2*^ *:Foxo1* ^*fl/fl*^ *v*essel which had severe back leak (Psn–Pin = 4.0 cmH_2_O) due the inability of the commissures on the right side to join. A gap (filled arrowhead) was always present regardless of the direction of the applied pressure gradient. The top image provides the projection acquired from imaging (without rotation) and its corresponding scale bar. See the explanation in Figure 1. See Supplementary Movie S9 for a rotatable 3D view of this structure.

One example of a mature but leaky valve from a *Prox1CreER* ^*T2*^ *:Foxo1* ^*fl/fl*^ mouse is shown in Figure 6. The morphology of this stage 4 valve appeared normal under brightfield illumination, but when tested for back leak, it was found to be completely leaky (back leak = 4.0 cmH_2_O). Subsequent live imaging of the valve (without fixation) under the confocal microscope revealed that the commissures on one side (the right sides of the middle and lower images, marked by arrowheads in the middle and lower panel) failed to join. Under an adverse pressure gradient, as shown here, a significant gap between the leaflets was always present on that side. Whether the same explanation applied to the other two leaky stage 4 valves (Figure 4) observed in *Prox1CreER* ^*T2*^ *:Foxo1* ^*+/fl*^ mice was not determined.

## Discussion

The images in Figure 2 are the first 3D images of LVs in various stages of development, and our study is the first attempt to correlate the developmental stage of the LV with an index of its physiological function (back leak). Our results support the four major stages of LV development described in other studies (Bazigou et al., 2009; Sabine et al., 2012; Bazigou and Makinen, 2013) and confirm that the geometry depicted in a summary drawing of murine venous valves by Bazigou et al. (Figure 2 in (Bazigou et al., 2011)] generally applies to murine LVs.

We justify the proposed addition of an intermediate stage of LV development (stage 3.5) based both on morphological appearance and functional measurements. Stage 3.5 valves were oriented with their openings angled toward one edge of the wall, and those openings were seldom visible in brightfield images, even after rotation, which is one reason why we could not provide images of such valves in a previous study (Scallan et al., 2021). Although stage 3 valves were uniformly leaky (Figure 4), most stage 3.5 valves had normal levels of back leak (<0.2 cmH_2_O); however, a subpopulation (16%) had varying degrees of elevated back leak, up to and including complete incompetence (3.7 cmH_2_O). No stage 4 valves in control mice (*Foxo1* ^*fl/fl*^) or *Foxc2CreER* ^*T2*^ *:Foxo1* ^*fl/fl*^ mice were this leaky, and only a single *Foxo1* ^*fl/fl*^ valve at stage 4 had even modest back leak (1.3 cmH_2_O). The latter equates to 2/22 (9%) of stage 4 valves from *Foxo1* ^*fl/fl*^ mice that were slightly leaky (>0.3 cmH_2_O) and is consistent with ∼10% of slightly leaky control valves that we have reported in other studies (Lapinski et al., 2017; Munger et al., 2017; Davis et al., 2023). The slight leak in those valves may reflect the higher turnover of LECs in valve leaflets compared to the rest of the vessel (10% vs. 1%), as reported recently (Saygili Demir et al., 2023), and implies that valve function may be temporarily compromised to a slight degree during the turnover of critical LECs.

A general pattern emerged from our analyses. Stage 1 valves were completely leaky, stage 2–3 valves had intermediate-to-severe leak, and most stage 3.5–4 valves were not leaky at all. For stage 3.5 valves, all (3 of 3) valves induced using *Foxc2CreER* ^*T2*^ were tight, and half (3 of 6) of the valves induced using *Prox1CreER* ^*T2*^ were tight, but 2 of 3 *Prox1CreER* ^*T2*^ *:Foxo1* ^*fl/fl*^ and 1 of 3 *Prox1CreER* ^*T2*^ *:Foxo1* ^*+/fl*^ stage 3.5 valves had intermediate-to-severe back leak. Among stage 4 valves, all (9 of 9) *Foxc2CreER* ^*T2*^ *:Foxo1* ^*fl/fl*^ valves were tight and most *Prox1CreER* ^*T2*^ *:Foxo1* ^*fl/fl*^ and *Prox1CreER* ^*T2*^ *:Foxo1* ^*+/fl*^ were tight, but 1 of 14 *Prox1CreER* ^*T2*^ *:Foxo1* ^*fl/fl*^ valves (Figure 6) and 2 of 14 *Prox1CreER* ^*T2*^ *:Foxo1* ^*+/fl*^ *valves* had severe back leak. One possible explanation is that the stage 3.5 valves from *Foxc2CreER* ^*T2*^ mice were at a slightly more advanced stage than the stage 3.5 valves from *Prox1CreER* ^*T2*^ mice. Although we could discern no obvious morphological differences between leaky and tight 3.5 valves (but we imaged only a few of the former), it is possible that early stage 3.5 valves are leaky and late stage 3.5 valves are not; however, 11% (3 of 28) of stage 4 valves in *Prox1CreER* ^*T2*^ mice were also leaky, suggesting that defects remain after the valves mature further. Thus, a second explanation for these results is that Foxo1 levels need to be restricted or titrated for proper development of new LVs and that *Foxc2CreER* ^*T2*^ is more effective than *Prox1CreER* ^*T2*^ for this process. However, we also cannot rule out the potential influence of *Foxc2* hemizygosity when using *Foxc2CreER* ^*T2*^. Staining for laminin-α5, integrin-α9, and other late stage LEC markers (Bazigou and Makinen, 2013) could possibly aid in distinguishing between stage 3, 3.5, and 4 valves. Additional genetic analyses of individual valves, such as PCR and ChIP-Seq, would also be informative, but are not practical in this context as the delicate single-valve vessel segments contain relatively few cells and are easily lost in processing.

Stage 3.5 valves induced by *Foxo1* deletion with *Foxc2CreER* ^*T2*^ were uniformly tight (Figure 4). However, the openings of these valves were not oriented along the centerline of the vessel like fully mature, stage 4 valves nor had symmetrical sinuses developed at stage 3.5 (Figure 5, Columns 3–4). Although these stage 3.5 valves may be effective in preventing the backflow of lymph (and pressure) in the face of adverse pressure gradients experienced *in vivo* (e.g., gravitational loads in human patients), their somewhat unusual morphology may have other consequences until they fully transition to stage 4. For example, the apparently smaller openings of stage 3 and 3.5 valves (Figure 5) may present an elevated resistance to forward flow in the open state, impeding lymph transport. A second consequence of the off-axis orientation of a stage 3.5 valve might be to alter the shear stress field at the leaflet tip/valve sinus, thus changing the normal flow pattern of lymph, which could affect the adhesion of lymphocytes and/or nitric oxide production. Given the importance that shear stress appears to have on lymphatic valve development, whether LECs are sensitive or responsive to the dynamic flow path over the course of valve development is an intriguing question. These possibilities remain to be tested either with experiments or modeling.

## Many questions remain unanswered about the processes underlying each stage of LV development

*Stage 1.* Oscillatory shear stress (OSS) is generally considered to be the normal initiator of LV formation (Sabine et al., 2012; Choi et al., 2019; Saygili Demir et al., 2023), and one effect of OSS is to alter *Foxo1* activity in the nucleus (Scallan et al., 2021). If the first step in valve formation is the emergence of a valve-forming cluster of *Prox1+* endothelial cells into a ring-shaped valve territory, is the expression of LEC polarity molecules (perhaps when Foxo1 leaves the nucleus) one of the early steps in LV formation? Does the ring of LECs in stage 1 start at a single point and spread circumferentially in both directions? Or can it start at more than one site, as suggested by the image in Figure 2, and, if so, what signals coordinate the expansion of two segments so that they eventually join?
*Stages 2–3.* Sema3A is known to control LEC migration, such that *Sema3A* ^*−/−*^ mice have a high percentage of valves with shorter leaflets (Bouvree et al., 2012). Is Sema3A–PlexinA1 signaling involved in stimulating the formation of the annulus and, once it begins to form, what stops the LEC migration (or turns off Sema3A production) to prevent full closure of the lumen? After LECs in one side of the “mature” annulus migrate downstream, how are LECs in the exact opposite side of the annulus signaled to later migrate downstream? Are they guided by deposition of FN-EIIIA, the deletion of which appears to abort valve extension at this stage (Bouvree et al., 2012)? Do LECs in these early LV stages communicate with each other through both paracrine and intercellular signaling since valves lacking the LEC–LEC gap junction protein Cx43 also have abnormally short leaflets (Munger et al., 2017)?
*Stage 3–3.5.* What is the composition of the commissure? Not only is this structure enriched in VE-cadherin (Figure 1), but valve degeneration is an early consequence of postnatal VE-cadherin deletion (Hagerling et al., 2018; Yang et al., 2019). It would be informative to test valve function in stage 4 LVs over time after VE-cadherin deletion. Are other tight junction molecules also needed to form what is apparently the strongest structure in the vessel—a structure capable of deforming to the extent that it can resist extreme adverse pressure (up to a 100 cmH_2_O gradient) without involuting (Lapinski et al., 2017)?
*Stage 4.* LMC coverage in the valve sinus is sparser than in non-valve regions [Figure 1 and (Petrova et al., 2004)], and reduced LMC density in the sinus is controlled during development by an interaction between Sema3A produced by LECs and NRP-1 receptors on LMCs (Bouvree et al., 2012; Jurisic et al., 2012). Moreover, the sinus wall is more distensible in this region than other parts of the vessel, which may facilitate normal valve gating (Bertram and Davis, 2023). Our results suggest that the sinus begins to remodel between stages 3 and 3.5 (Figures 1, 5) when the first commissure is extending into the wall. If so, is there something in the process of commissure insertion that triggers Sema3A production?

Ultimately, the development of an *in vitro* LEC culture system with controlled laminar and/or oscillatory shear stress to monitor and record LV development in real time, and with the capability to modify critical genes as needed via siRNA knockdown, will provide more definitive information about the mechanisms of LV development. In the meantime, the techniques applied to developing LVs in this study can potentially be used to answer these questions, particularly if they can be combined with spatial scRNA-seq methods to identify which valve LECs are expressing certain genes at critical time points.

## Data Availability

The original contributions presented in the study are included in the article/Supplementary Materials; further inquiries can be directed to the corresponding authors.

## Supplementary material

SUPPLEMENTARY MOVIE S1 Stage 4 valve (from Figure 1) rotated through various positions.

SUPPLEMENTARY MOVIE S2 Stage 4 valve (from Figure 1) rotated through various positions.

SUPPLEMENTARY MOVIE S3 Stage 3 valve (from Figure 1) rotated through various positions.

SUPPLEMENTARY MOVIE S4 Stage 1 valve (from Figure 2) rotated through various positions.

SUPPLEMENTARY MOVIE S5 Stage 2 valve (from Figure 2) rotated through various positions.

SUPPLEMENTARY MOVIE S6 Stage 3 valve (from Figure 2) rotated through various positions.

SUPPLEMENTARY MOVIE S7 Stage 3.5 valve (from Figure 2) rotated through various positions.

SUPPLEMENTARY MOVIE S8 Stage 4 valve (from Figure 2) rotated through various positions.

SUPPLEMENTARY MOVIE S9 Leaky stage 4 valve (from Figure 6) rotated through various positions.
